# Australian Digital Mental Health Services for Anxiety and Depression: Meta-Analysis and Comparison Study

**DOI:** 10.2196/90121

**Published:** 2026-10-06

**Authors:** Bridget Bassilios, Katrina J Scurrah, Amy J Morgan, Leo Roberts, Maria Ftanou, Matthew J Spittal

**Affiliations:** 1 Centre for Mental Health and Community Wellbeing, Melbourne School of Population and Global Health, The University of Melbourne, Victoria, 3010, Australia, 61 3 8344 0662

**Keywords:** digital mental health services, depression, anxiety, online therapy, e-therapy, telehealth, clinician-supported, self-guided

## Abstract

**Background:**

Mental Health Online, MindSpot, and THIS WAY UP are 3 Australian Government–funded digital mental health services (DMHSs). These services are free for consumers and deliver a range of (depression and anxiety disorder) psychological assessments and interventions using the internet, with or without clinician support.

**Objective:**

This study examines the uptake and effectiveness of these services.

**Methods:**

We used 3 data sources to examine the performance of the three DMHSs: (1) aggregated routinely collected service data to describe uptake of the services from January 2013 to December 2021, (2) peer-reviewed and gray literature reporting treatment outcomes, and (3) treatment outcome data from one evaluation conducted by Mental Health Online. The second and third data sources were used to analyze the effectiveness of the DMHSs compared to other mental health treatment programs (Australian primary, public, and low-intensity mental health care; UK stepped psychological care). We generated pooled mental health symptom (Cohen *d*) effect sizes for each service’s clinician-supported and self-directed treatments and compared those with effect sizes we calculated for the alternative treatment programs.

**Results:**

The 3 DMHSs offered care to more than 282,000 consumers and therefore contributed to improving overall access to mental health care in Australia. Clinician-supported online treatment significantly improved the mental health of consumers who use these services (Mental Health Online, Cohen *d*=0.95; MindSpot, Cohen *d*=1.42; and THIS WAY UP, Cohen *d*=1.04), and self-directed treatment by Mental Health Online produced a moderate reduction (Cohen *d*=0.59) in anxiety disorder severity ratings. Clinician-supported treatments produced improvements in mental health symptoms that are close or equivalent to most (face-to-face, phone, and stepped or symptom severity–matched mental health care) comparison treatments examined.

**Conclusions:**

Mental Health Online, MindSpot, and THIS WAY UP are producing clinically significant improvement for consumers experiencing psychological distress and anxiety and depression symptoms. The magnitude of improvement produced by clinician-supported treatment is comparable with more resource-intensive face-to-face treatment options. Heterogeneity in the magnitude of improvements produced by the 3 DMHSs (and comparator interventions across levels of clinical need) indicates that findings should be interpreted with caution, particularly in relation to the populations for whom DMHSs may be most appropriate.

## Introduction

Globally, mental disorders are among the top 10 leading causes of disease burden [1]. From 2020 to 2022 in Australia, 21.5% of adults experienced a mental disorder each year, of whom 45.1% saw a health professional and 4.8% accessed digital technologies for their mental health [2]. Although the proportion of people accessing services for their mental health has increased by more than 10% (35% in 2007 [3]), there is potential to further improve service access by using digital mental health services (DMHSs).

DMHSs remotely deliver psychological interventions by phone (eg, crisis and counseling services), videoconference-based connections (eg, telehealth), or online (desktops, mobile devices, and apps) [4,5]. DMHSs can help improve access to mental health care and/or complement traditional face-to-face care given their scalability and the ubiquity of desktop, mobile, and telephone devices. DMHSs are low-cost for consumers and have the potential to reach people who do not or cannot access traditional services (eg, people in rural, remote, or low-income regions) in a convenient setting (home, workplace, school, community, or clinical service). DMHSs have the added advantage of reducing the stigma associated with using mental health services by offering users anonymity and the ability to manage their mental health problems in real-time, 24 hours a day, 7 days a week [6].

Digital mental health interventions (DMHIs) or treatments are one type of DMHS offering. DMHIs may be self-directed (unguided) or human-supported (guided). In the case of the latter, support may be provided by therapists (clinicians), volunteer crisis supporters, teachers, administrators, or peers [5]. Support may include monitoring and/or optional support completing self-directed online modules or therapist support, typically occurring after consumers complete successive online modules. A systematic meta-review of 31 meta-analyses (505 unique primary studies; largely randomized controlled trials [RCTs]) reported that human-supported DMHIs were more effective than self-directed DMHIs [7]. However, the authors were tentative about those findings because of significant heterogeneity observed across studies. Nonetheless, 9% (4/45) of studies showed that self-directed DMHIs were significantly more effective than treatment as usual. Additionally, the authors called for future research to provide detailed descriptions of sample and intervention characteristics, as well as the mechanism through which human support is most useful for the given DMHI—a need that this study at least partially addresses.

In recognition of the potential for DMHSs to improve access to, and outcomes of, mental health care, the Australian Government has been funding various DMHSs since 2006 when the Teleweb (Telephone Counseling, Self Help, and Web-based Support Programs) Measure was introduced. The Australian Government Productivity Commission Mental Health Inquiry Report specifically noted the potential benefits of clinician-supported DMHSs [8], which are listed on the Australian Government mental health platform, Head to Health [9] (now known as “Medicare Mental Health”; Liquid Interactive).

Three major Australian Government–funded DMHSs offering online treatment with the option of clinician support are Mental Health Online (National eTherapy Centre, Swinburne University), MindSpot (MQ Health, Macquarie University), and THIS WAY UP (St Vincent’s Hospital, University of New South Wales). These DMHSs have been operating for over a decade and provide services at no cost to consumers (with the exception that THIS WAY UP charged a small fee before 2019). All 3 DMHSs offer web-based information, online assessments including risk management and service navigation, and self-directed and clinician-supported cognitive behavioral therapy (CBT)–based treatment (in 3‐12 sessions or modules), mainly for adults with depression and anxiety disorders [10]. However, their service delivery models differ from one another and so does the amount of government funding they each receive. For example, clinician support is provided by internal provisional (trainee) psychologists at Mental Health Online, internal allied health clinicians at MindSpot, and the consumer’s own health professional externally at THIS WAY UP. Although clinician support is available, consumers can choose not to take up this support and instead elect to complete online treatment on their own (self-directed treatment).

This study was conducted as part of a larger mixed methods independent evaluation [10], which also included an environmental scan of DMHSs, an umbrella literature review of the efficacy of DMHIs, and various stakeholder consultations—the findings from which are detailed elsewhere [11-13]. Briefly, the environmental scan used the RE-AIM (Reach, Efficacy or Effectiveness, Adoption, Implementation, and Maintenance) framework to evaluate Australian DMHSs [14]. It showed that (1) DMHSs have been reaching a steadily increasing number of consumers since at least 2014, with demand increasing for clinician-supported DMHSs since the COVID-19 pandemic; (2) DMHSs involving support or guidance lead to positive mental health outcomes and have the potential to be good value for money, especially for adults with depression and anxiety disorders; and (3) there are opportunities to improve reach and adoption, determine effectiveness in subpopulations, and make optimal use of technology [11]. Consistent with the literature, our umbrella review found that clinician-supported DMHIs are effective for treating depression and anxiety disorders in adults [12]. Among other findings, the overall evaluation showed that despite occasional potential difficulties (technical and rapport-building), both consumers and providers are largely satisfied with receiving and delivering care via Australia’s 3 key DMHSs, and DMHSs are cost-effective compared to usual care [10,13].

Complementing our umbrella review, which used trial data to examine whether DMHSs can work under ideal, controlled conditions, this study examined whether DMHSs work in routine real-world settings, using uptake and outcome data from Mental Health Online, MindSpot, and THIS WAY UP. Importantly, this study also compares the magnitude of the self-directed and clinician-supported outcomes produced by these 3 DMHSs to other key mental health treatments available in the Australian context and the United Kingdom, including other digitally delivered options and traditional, more resource-intensive face-to-face options. The study may contribute to international policy decisions specifically related to investment in clinician-supported and self-directed DMHSs.

## Methods

### Ethical Considerations

Ethics approval was granted by The University of Melbourne’s Human Research Ethics Committee (ID: 22376). Each DMHS collected administrative service use data using its own consent process. The data analyzed in this study were provided by each DMHS in aggregate form and excluded potentially identifying individual information.

### Data Sources

#### Overview

This study used 3 data sources to examine the performance of the 3 DMHSs—routinely collected service use data, peer-reviewed and gray literature, and outcome data from a one-off evaluation conducted by Mental Health Online that was not publicly available.

#### Routine Service Use Data

The 3 DMHSs routinely collect administrative service use data. We requested aggregate uptake data from each DMHS split by service pathway (ie, assessment, self-directed treatment, and clinician-supported treatment). Mental Health Online data were from January 2015 to December 2021; MindSpot, January 2013 to December 2021; and THIS WAY UP, July 2015 to December 2021. The purpose of these data was to provide a context for the analyses of effectiveness of the 3 DMHSs.

There are similarities and differences in the data item types and frequency in which they are collected. For example, MindSpot and THIS WAY UP collect mental health outcome data at each session whereas Mental Health Online collects outcome data during baseline assessment, preimplementation program trials, and discrete evaluations.

The 3 DMHSs use a variety of standardized outcome measures to assess mental health and well-being outcomes for consumers, most commonly the Kessler Psychological Distress 10-Item Scale (K10) [15] measuring psychological distress, the Patient Health Questionnaire-9 (PHQ-9) [16] measuring depression symptoms, and the Generalized Anxiety Disorder 7-item scale (GAD-7) [17] measuring anxiety symptoms (MindSpot and THIS WAY UP), with the Kessler 6-item version (K6) [18,19] used at baseline only (Mental Health Online). Multimedia Appendix 1 describes each of these and other measures used by the 3 DMHSs.

#### Peer-Reviewed and Gray Literature

##### DMHSs

For MindSpot and THIS WAY UP, we included peer-reviewed publications on their effectiveness that:

- focused on real-world evaluation of their mental health treatment programs (rather than RCTs) overall and for disadvantaged groups
- provided data on a range of outcomes (not just mental health outcomes)
- reported whether treatment was clinician-supported or self-directed
- were the most recently published, included the largest sample size, or provided outcomes by disorder or program type.

However, because Mental Health Online does not routinely collect outcome data, we included peer-reviewed preimplementation studies (various designs) for this service.

We selected these publications from a detailed service description document prepared for us by MindSpot [20] and from extensive lists of peer-reviewed publications supplied by all 3 DMHSs. Data on study characteristics and treatment outcomes were then extracted by AJM (and checked by BB) from the selected publications and entered in a Microsoft Excel spreadsheet. Specific data types extracted included authors, publication year, aim, sample size and characteristics, recruitment methods, study design, intervention length, and description including involvement of clinician support, and types of standardized outcome measurement tools used including baseline and posttreatment score means and SDs.

##### Comparison National and International Programs or Trials

We also purposively included peer-reviewed and gray literature on other key, low-to-high-intensity mental health treatments available in Australia for which outcome data are routinely collected and reported; and a relevant UK program (providing similar services to the Australian DMHSs through its low-intensity component) for comparison. Some of these publications were our own evaluations of Australian national primary mental health programs (eg, Better Access, Access to Allied Psychological Services, and the Link-me RCT) and the UK stepped mental health care Improving Access to Psychological Therapies (IAPT) program (now known as Talking Therapies), which provides low- and high-intensity intervention matched to individual level of need (symptom severity) [21,22]. Other comparators were requested by the Department of Health (eg, New Access and a low-intensity treatment involving telephone coaching for people with mild-to-moderate symptoms) or the DMHSs (eg, public sector intended for people with relatively more severe and complex needs). Using an Excel spreadsheet to input the data, BB extracted (and KJS checked) authors or data source, sample sizes, design, intervention characteristics, and types of standardized outcome measurement tools used, including baseline and posttreatment score means and SDs. The standardized outcome measurement tools used by the comparator programs are described in Multimedia Appendix 1.

### Data From an Evaluation of Mental Health Online

Mental Health Online only collects pre- and posttreatment mental health outcome data for discrete evaluations of various service aspects. Therefore, mental health outcomes collected during one such evaluation from September 2, 2021 to February 2, 2022, were directly provided by Mental Health Online and included in our effectiveness analyses. Details about how Mental Health Online collected these data and their analysis indicating this sample’s representativeness of their wider group of consumers (n=2318) of the clinician-supported (Therapist Assist) program are reported elsewhere [10].

### Data Analysis

We conducted descriptive analyses of existing routinely collected service use data provided in aggregate form by the 3 DMHSs to report on uptake by service type (assessment or treatment) and treatment type (clinician-supported or self-directed).

To examine the effect of treatment on mental health symptoms or functioning, we imported into Stata (StataCorp LLC) quantitative data from publications on the 3 DMHSs and the comparator programs (sample sizes, means and SDs at baseline and posttreatment, and whether the treatment was self-directed or clinician-supported; supplemented by additional evaluation data from Mental Health Online). Decreases in scores for the original measures of symptoms and disability, and increases in scores for measures of quality of life and functioning, indicated improvement. Because different measures were frequently used, we calculated the effect size as a standardized mean difference (specifically, Cohen *d*). This enabled us to compare pre- and posttreatment means for specific groups using a common metric. Cohen [23] recommends interpreting effect sizes of Cohen *d*=0.2 as “small,” 0.5 as “medium,” and 0.8 as “large” effect sizes. For ease of interpretation, all effect size estimates were reported as positive if posttreatment means showed improvements compared with pretreatment means, and negative if posttreatment means showed deterioration compared with pretreatment means.

Next, we generated a pooled effect size for each service’s clinician-supported and self-directed treatments. The pooled mental health effect sizes were compared with effect sizes we calculated for comparison treatments. Individual effect sizes were pooled using a random-effects meta-analysis and estimated using restricted maximum likelihood. All analyses were undertaken in Stata (v16; StataCorp LLC) [24].

## Results

### Uptake of DMHSs

Table 1 summarizes key uptake data for the 3 DMHSs across different periods.

**Table 1.** Summary uptake statistics.

Service type	Mental Health Online (January 2015- December 2021)	MindSpot (January 2013- December 2021)	THIS WAY UP (July 2015- December 2021)
Started assessment, n	—^a^	171,070	149,002
Completed assessment, n	24,495	133,447	124,270^b^
Enrolled in treatment, n	17,916	35,942	72,007
Started treatment	—	30,384	54,510
Completed treatment^c^	—	20,267	24,989
Enrolled in clinician-supported treatment	1011	34,390	34,048
Started or allocated clinician-supported treatment	2319^d^	28,832	27,405
Completed clinician-supported treatment^c^	463^e^	18,715	13,431
Enrolled in self-directed treatment	16,905	1552^f^	37,959
Started self-directed treatment	—	1552^f^	27,105
Completed self-directed treatment^b^	—	1552^f^	11,558

a Not available.

b Stage 1 assessment (initial anonymous online questionnaire); 71,069 stage 2 assessment (clinician-supported assessment).

c Completion defined as the completion of ≥6 of 12 weeks for Mental Health Online, ≥4 lessons for MindSpot, and two-thirds or more of lessons for THIS WAY UP.

d Includes 1308 consumers registered for self-directed treatment.

e A total of 463 of 815 consumers who were allocated a clinician from July 2018 to August 2021.

f Self-directed treatment introduced in July 2019.

Over 7 years from January 2015 to December 2021, 24,495 consumers completed an assessment (with or without treatment) with Mental Health Online. Overall, 17,916 consumers (who had or had not completed an assessment) enrolled in a treatment program, 2319 (13%) of whom registered to receive clinician support. From July 2018 to August 2021 (period during which session attendance was being documented in a readily collatable dataset), 815 consumers were allocated a clinician, 57% (463) of whom completed supported treatment.

For MindSpot over 9 years from January 2013 to December 2021, 133,447 assessments were completed and 27% (35,942) of consumers who completed an assessment enrolled in either clinician-supported or self-directed treatment programs. This should be interpreted in the context that around 67% (65,042/97,127) of MindSpot consumers report that an assessment is their primary need [25]. In the overall period of data provision, most consumers enrolled in clinician-supported treatment (34,390/35,942, 96%). From July 2019, when self-directed treatment was introduced, to December 2020, 83% (7597/9149) of consumers enrolled in clinician-supported treatment. Two-thirds of those who started clinician-supported treatment completed treatment.

Data from THIS WAY UP show that over 6 and a half years from July 2015 to December 2021, 149,002 consumers started the assessment, and 124,270 stage 1 (initial anonymous online questionnaire) and 71,069 stage 2 (clinician-supported) assessments were completed. Approximately 72,000 consumers enrolled in any treatment and 34,048 (47%) in clinician-supported treatment. Three-quarters of all enrollments (54,510/72,007) started treatment. Of consumers who started, 46% (24,989/54,510) completed treatment (ie, at least two-thirds of lessons).

Of consumers who enrolled in MindSpot and THIS WAY UP treatments, 85% (30,384/35,942) and 76% (54,510/72,007), respectively, commenced treatment (equivalent data were not available for Mental Health Online).

### Effectiveness of DMHSs

#### Included Studies and Data

##### DMHSs

Table 2 describes the key characteristics of DMHS studies and data (Mental Health Online, unpublished) included in our effectiveness analyses.

**Table 2.** DMHS^a^ studies and data included in effectiveness analyses.

Study or data	Sample description and size	Recruitment	Design	Intervention	Clinician support available	Measures
Mental Health Online						
Unpublished data (collected and directly provided by MHO^b^)	Adults (n=25)	MHO service users from September 2021 to February 2022 Depression Online (n=6) Made4Me (transdiagnostic course; n=6) GAD^c^ Online (n=5) Panic Stop (n=3) OCD^d^ Stop (n=2) PTSD^e^ Stop (n=2) SAD^f^ Online (n=1)	One-off evaluation, pre-post quasi-experimental, clients received a US $7 ($10 AUD) gift card per outcome measurement occasion	12-week iCBT^g^	Yes	K6^h^
Klein et al (2011) [26]	225 adults with at least subclinical levels of anxiety disorders GAD (n=88) PD^i^ (n=40) OCD (n=17) PTSD (n=30) SAD (n=50)	Anxiety Online (now MHO) service users. International public recruited via Facebook advertisements, referral links on mental health websites, media, presentations, and mailouts to health professionals and consumer groups	Pre-post quasi-experimental	Five 12-week iCBT modules (for GAD, PD, OCD, PTSD, and SAD)	No	K6 Clinical disorder severity rating (GAD, PD, OCD, PTSD, SAD) Quality of Life
Klein et al (2010) [27]	Adults with PTSD (measured by the clinician-administered PTSD Scale for *DSM IV*^j^) (n=89)	Recruited via Australian mental health websites and media	Pre-post quasi-experimental	10-week iCBT for PTSD	Yes (mean 194.5 min)	PTSD clinician severity rating PCL-C^k^ WHO-QOL-BRF^l^ (psych)
Kyrios et al (2018) [28]	Adults with OCD (n=89)	Referred by primary care physicians and mental health professionals and through self-referral. Advertisement (webpage, affiliated online mental health treatment webpage, YouTube, Facebook, mail-outs to Australian mental health professionals).	RCT^m^	12-week iCBT for OCD	Yes	YBOCS^n^
MindSpot						
Kayrouz et al (2020) [29]	Non–Australian-born adults with anxiety and/or depression NESB^o^ MidEast (n=43) NESB Europe (n=115) NESB Asia (n=182) NESB English (n=323) ESB^p^ English (n=930)	MindSpot (Wellbeing course) service users from January 2014 to December 2016	Retrospective observational cohort, pre-post quasi-experimental design	8-week (5-module) iCBT Wellbeing Course	Yes	PHQ-9^q^ GAD-7^r^
Staples et al (2019) [30]	Young people (18‐24 years) with anxiety or depression (n=222)	MindSpot Mood Mechanic (transdiagnostic course for young adults) service users from January to June 2016	Retrospective pre-post quasi-experimental	8-week (5-module) iCBT Mood Mechanic (transdiagnostic) course	Yes	K10^s^ PHQ-9 GAD-7
Staples et al (2016) [31]	Older adults (aged ≥60) with anxiety or depression (n=516)	MindSpot Wellbeing Plus (transdiagnostic course for adults aged ≥60 years) service users from January 2013 to June 2015	Observational real-world pre-post quasi-experimental	8-week (5-module) iCBT Wellbeing Plus Course	Yes	K10 PHQ-9 GAD-7
Titov et al (2020) [25]	Adults with anxiety or depression (n=21,745)	MindSpot service users from January 2013 to December 2019	Observational pre-post quasi-experimental	8-week (5-module), one of 7 iCBT courses (4 transdiagnostic for anxiety and depression; and 3 disorder-specific—OCD, PTSD, and chronic pain)	Yes	K10/K10+^t^ PHQ-9 GAD-7
Titov et al (2019) [32]	Indigenous adults with anxiety or depression (n=70)	MindSpot (standard Wellbeing or Indigenous Wellbeing course) service users from January 2015 to December 2016	Prospective uncontrolled observational cohort	8-week (5-module) iCBT Indigenous Wellbeing Course	Yes	K10 PHQ-9 GAD-7
THIS WAY UP						
Allen et al (2016) [33]	Adults with PD (n=330)	Primary care patients prescribed the Clinical Research Unit for Anxiety and Depression (CRUfAD^u^; now TWU^v^) Panic Program from August 2011 to June 2014	Observational pre-post quasi-experimental	5-module iCBT for PD (to be completed within 90 days)	Yes	K10 PHQ-9 PDSS-SR^w^ WHODAS 2.0^x^
Hobbs et al (2018) [34]	Adults with depression (n=586) 18‐24 years (n=44) 25‐34 years (n=105) 35‐44 years (n=149) 45‐54 years (n=135) 55‐64 years (n=108) ≥65 years (n=45)	Patients prescribed TWU iCBT for depression by their clinician from May 2009 to October 2015	Observational real-world pre-post quasi-experimental	6 modules of iCBT for depression	Yes	K10 PHQ-9 WHODAS 2.0
Hobbs et al (2017) [35]	Adults with GAD 18‐29 years (n=100) 30‐39 years (n=117) 40‐49 years (n=82) 50‐59 years (n=77) ≥60 years (n=65)	Patients prescribed and completed at least one session of TWU iCBT for GAD from March 2012 to November 2015	Observational pre-post quasi-experimental (part of routine quality assurance activities of TWU)	6 modules of iCBT for GAD (to be completed within days)	Yes	K10 PHQ-9 PDSS-SR WHODAS 2.0
Newby et al (2017) [36]	Adults with depression or anxiety (n=1005)	Patients prescribed TWU iCBT course by their clinician (GP^y^, psychologist, nurse, or other allied health professional)	Observational real-world pre-post quasi-experimental (part of routine quality assurance activities of CRUfAD)	6 modules of transdiagnostic iCBT	Yes	K10 GAD-7 PHQ-9 WHODAS 2.0
Williams et al (2014) [37]	Adults with SAD (n=368)	Patients prescribed TWU iCBT course by their clinician from September 2010 to February 2014	Observational real-world pre-post quasi-experimental	6 modules of iCBT for SAD (to be completed within 90 days)	Yes	K10 PHQ-9 Mini-Spin^z^ WHODAS 2.0

a DMHS: digital mental health service.

b MHO: Mental Health Online.

c GAD: generalized anxiety disorder.

d OCD: obsessive-compulsive disorder.

e PTSD: posttraumatic stress disorder.

f SAD: social anxiety disorder.

g iCBT: internet-based cognitive behavioral therapy.

h K6: Kessler Psychological Distress 6-Item Scale [18,19].

i PD: panic disorder.

j *DSM-IV: Diagnostic and Statistical Manual of Mental Disorders, Fourth Edition*.

k PCL-C: Post-traumatic Stress Disorder Checklist [38].

l WHO-QOL-BRF (psych): World Health Organization Quality-of-Life Scale Psychological Domain subscale [39].

m RCT: randomized controlled trial.

n YBOCS: Yale Brown Obsessive Compulsive Scale [40].

o NESB: migrant of a non–English-speaking background.

p ESB: migrant of an English-speaking background.

q PHQ-9: Patient Health Questionnaire-9 [16].

r GAD-7: Generalized Anxiety Disorder 7-item scale [17].

s K10: Kessler Psychological Distress 10-Item Scale [15].

t K10+: Kessler Psychological Distress 10 Plus-Item Scale [41].

u CRUfAD: Clinical Research Unit for Anxiety and Depression.

v TWU: THIS WAY UP.

w PDSS-SR: Panic Disorder Severity Scale Self-Report version [42].

x WHODAS 2.0: World Health Organization Disability Assessment Schedule [43].

y GP: general practitioner.

z Mini-SPIN: Mini-Social Phobia Inventory [44].

We included 3 peer-reviewed publications of Mental Health Online program trials preceding real-world routine implementation [26-28]. Two were uncontrolled trials and one was an RCT. Mental Health Online also provided us with data collected from 25 clinician-supported service users as part of a one-off evaluation. All data were collected from adults who had completed 10‐ to 12-week courses for anxiety disorders and/or depression.

We included 5 peer-reviewed publications of routine MindSpot treatment outcomes [25,29-32]. One of these studies provided pre- and posttreatment outcomes of routine clinician-supported treatment for consumers at each assessment point over MindSpot’s first 7 years of operation [25]. The remaining 4 studies complemented findings from this key publication by focusing on outcomes of routine clinician-supported treatment in younger and older adults, Indigenous peoples, and migrants [29-32]. Data from all 5 studies were collected from adults with anxiety and/or depression who enrolled in 5-module treatment courses.

We included 5 peer-reviewed publications of routine THIS WAY UP treatment outcomes covering outcomes from 5- or 6-module treatment courses across anxiety and/or depression disorders and adult age groups [33-37]. It should be noted that we classified all THIS WAY UP treatments as clinician-supported because THIS WAY UP encourages all registered clinicians to contact their patients at least twice during the course of completing an online program to maximize adherence and if their patient’s distress is high or increases between lessons [36,45].

Except for one Mental Health Online study of adults with anxiety disorders, clinician support was available (but not necessarily used by participants) in all studies on the 3 DMHSs. Sample sizes in all included data sources ranged from 25 (data provided by Mental Health Online) to 21,745 (MindSpot observational study). A range of outcome measures (Multimedia Appendix 1) were used in the studies, most commonly the K10 [15], PHQ-9 [16], and GAD-7 [17], to assess mental health outcomes; and quality of life and disability measures to assess wider well-being outcomes.

##### Comparison National and International Programs or Trials

Table 3 describes the national and international programs or trials included as comparators in our effectiveness analyses.

**Table 3.** Mental health outcomes produced by comparison national and international programs or trials.

Study or data source	Sample size	Sample description	Design	Intervention	Delivered by	Measures
Australian low-intensity mental health care (New Access) based on low-intensity IAPT^a^						
Baigent et al (2023) [46]	3579	≥18 years with mild-to-moderate anxiety and/or depression and not receiving treatment from psychologist or psychiatrist	Prospective cohort observational study with repeated measures using routinely collected service data from October 2013 to October 2016	Six coaching sessions provided over the phone (or face to face)	Six-week trained coaches with no requirement for prior mental health qualifications or clinical experience	PHQ-9^b^ GAD-7^c^
Australian primary mental health care						
Bassilios et al (2017) [47]	22,399	≥16 years mainly diagnosed with depression and/or anxiety disorders	Pre-post using routinely collected Tier 1 ATAPS^d^ data from July 2003 to June 2016	Up to 12 (or 18 in exceptional circumstances) individual face-to-face and/or 12 group sessions, with review by the referring GP^e^ after each block of 6 and/or the final session	Psychologists, social workers, mental health nurses, occupational therapists, or Aboriginal and Torres Strait Islander health workers	K10^f^
Pirkis et al (2011) [48]	193	Adults, >90% diagnosed with depression and/or anxiety disorder	Pre-post study using data from service users collected as part of an effectiveness evaluation from October 2009 to October 2010	Up to 12 (or 18 in exceptional circumstances) sessions of face-to-face psychological intervention, following development of a mental health care plan by a GP and with review by a GP at defined intervals	Clinical psychologists	K10
Pirkis et al (2011) [48]	192	As above	As above	As above	Psychologists	K10
Pirkis et al (2011) [48]	177	As above	As above	As above	GPs	K10
Australian treatment as usual control groups from Link-me RCT						
Fletcher et al (2021) [49]	416	Adults, mild depression or anxiety symptoms control group	Pre-post RCT^g^, data collected from November 2017 to October 2018	Patient-completed DST^h^ completed via tablet, to predict severity of depression or anxiety. Control group encouraged, on the tablet and via an automated email sent on completion of the Link-me DST, to discuss any mental health concerns with their GP. Participants were free to continue or modify any treatment they were receiving at trial entry, and to commence new or additional treatments at any time	No treatment or treatment as usual	K10
Fletcher et al (2021) [49]	427	Adults, moderate depression or anxiety symptoms control group	As above	As above	No treatment or treatment as usual	K10
Fletcher et al (2021) [49]	421	Adults, severe depression or anxiety symptoms control group	As above	As above	Care navigators (registered health practitioners) who worked as a clinical companion to the treating GP	K10
Australian public mental health outpatient care						
Australian Mental Health Outcomes and Classification Network (2022) [41]	144,288	Adults with severe mental disorders	Pre-post using routinely collected service data from July 2000 to June 2020	All nonadmitted, nonresidential services (ie, community-based crisis assessment and treatment teams, day programs, psychiatric outpatient clinics provided by either hospital or community-based services, child and adolescent outpatient and community teams, social and living skills programs, psychogeriatric assessment services, etc)	Health professionals with specialist mental health qualifications (eg, psychologists, mental health nurses, social workers, and psychiatrists)	K10
UK stepped mental health care (IAPT)						
NHS^i^ Digital (2021) [22]	595,840	Adults with depression and/or anxiety	Pre-post using routinely collected IAPT service data from April 2020 to March 2021	National Institute for Health and Care Excellence approved therapies for treating people with anxiety or depression. Includes face-to-face services, low-intensity services, group services, and self-directed book- or computer-based programs	Self-help, self-help guided by psychological well-being practitioners (low-intensity therapists), peer support workers, high-intensity therapists (nurses, clinical psychologists, social workers, occupational therapist, and others)	PHQ-9 GAD-7

a IAPT: Improving Access to Psychological Services.

b PHQ-9: Patient Health Questionnaire-9 [16].

c GAD-7: Generalized Anxiety Disorder 7-item scale [17].

d ATAPS: Access to Allied Psychological Services (Tier 1 refers to base funding or general ATAPS vs Tier 2 funding for hard-to-reach groups).

e GP: general practitioner.

f K10: Kessler Psychological Distress 10-Item Scale [15].

g RCT: randomized controlled trial.

h DST: Decision Support Tool.

i NHS: National Health Service.

Five key comparator categories were included. Four of these categories are Australian including (1) low-intensity mental health care (New Access) [46], (2) primary mental health care (Better Access [48] and Access to Allied Psychological Services [47]), (3) treatment as usual control groups from a RCT of Link-me [49] involving assessment of mental health symptoms among patients presenting for any health problem in primary care, and (4) adult ambulatory (outpatient) public mental health care [41]. The fifth category is UK-based stepped mental health care (IAPT) delivering a range of treatments matched to symptom severity ranging from low to high intensity [22].

Data were routinely collected from adults (aged ≥16 years) as part of service use except for participants in the Link-me RCT who were followed up as part of that study. Adults comprising the comparison groups experienced mainly depression and/or anxiety disorders ranging in severity from mild (eg, New Access and some IAPT consumers) to severe (public sector). The public sector category is most likely to have experienced other, including low prevalence, mental disorders (eg, schizophrenia and bipolar disorder).

Except for New Access, delivered by coaches over the phone, and some lower intensity IAPT services that are self-directed or supported by psychological well-being practitioners, most services are delivered face to face by health professionals.

Sample sizes ranged from 177 (Better Access) to around 596,000 (IAPT). Mental health outcomes are measured using the K10 [15], PHQ-9 [16], and/or GAD-7 [17].

### Effectiveness of DMHSs

Figures 1–3 show the mental health and quality-of-life outcomes produced by Mental Health Online, Mindspot, and THIS WAY UP, respectively.

**Figure 1.**
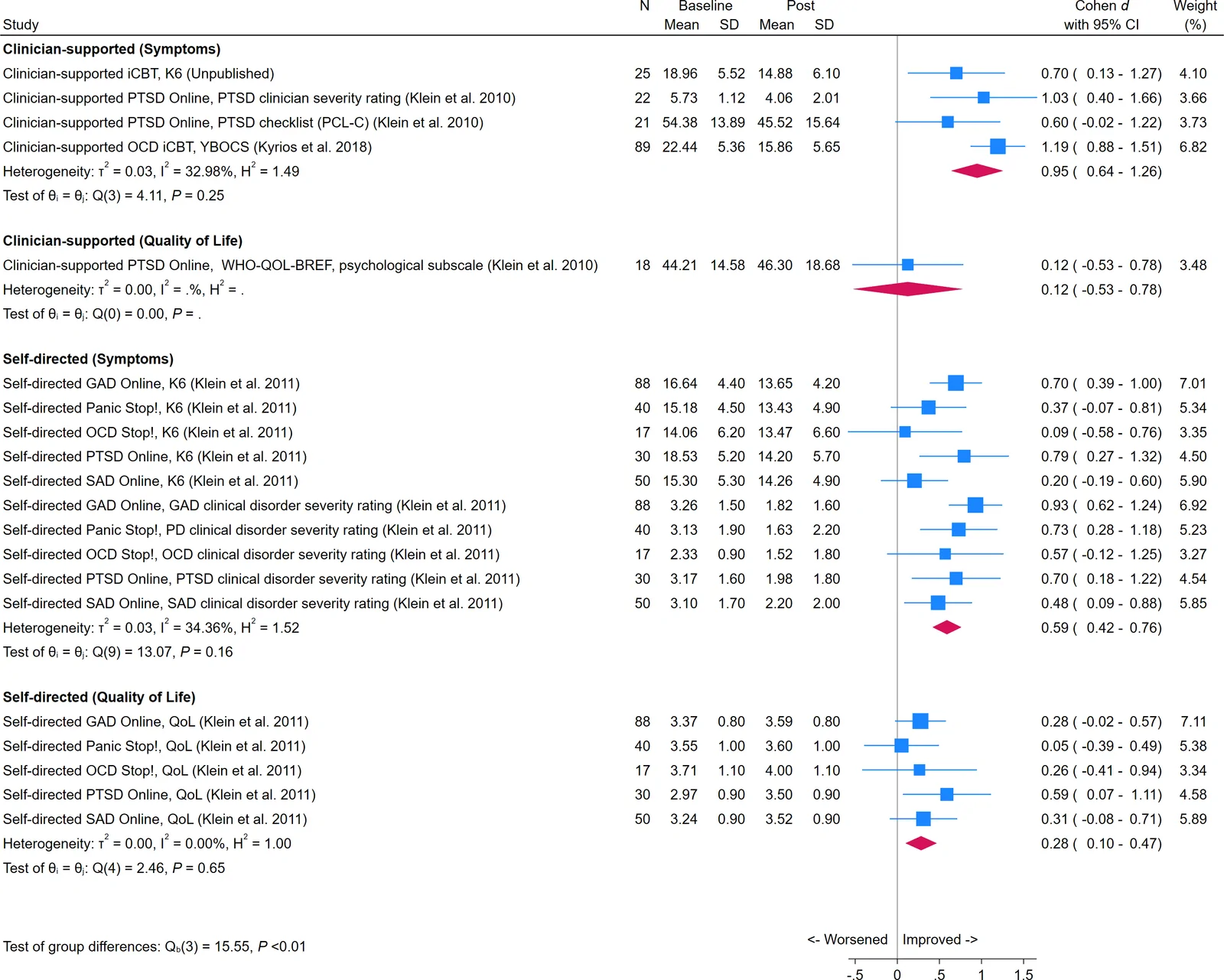
Forest plot of Mental Health Online (10‐12 weeks) cognitive behavioral therapy treatment effects on mental health symptoms and quality of life by treatment support type [26-28]. Better mental health and quality of life are represented by lower scores and higher scores, respectively. GAD: generalized anxiety disorder; iCBT: internet cognitive behavioral therapy; K6: Kessler Psychological Distress 6-Item Scale; OCD: obsessive-compulsive disorder; PCL-C: Posttraumatic Stress Disorder Checklist—Civilian Version; PD: panic disorder; PTSD: posttraumatic stress disorder; QoL: quality of life; SAD: social anxiety disorder; WHO-QOL-BREF: World Health Organization Quality of Life Questionnaire-BREF; YBOCS: Yale-Brown Obsessive Compulsive Scale.

**Figure 2.**
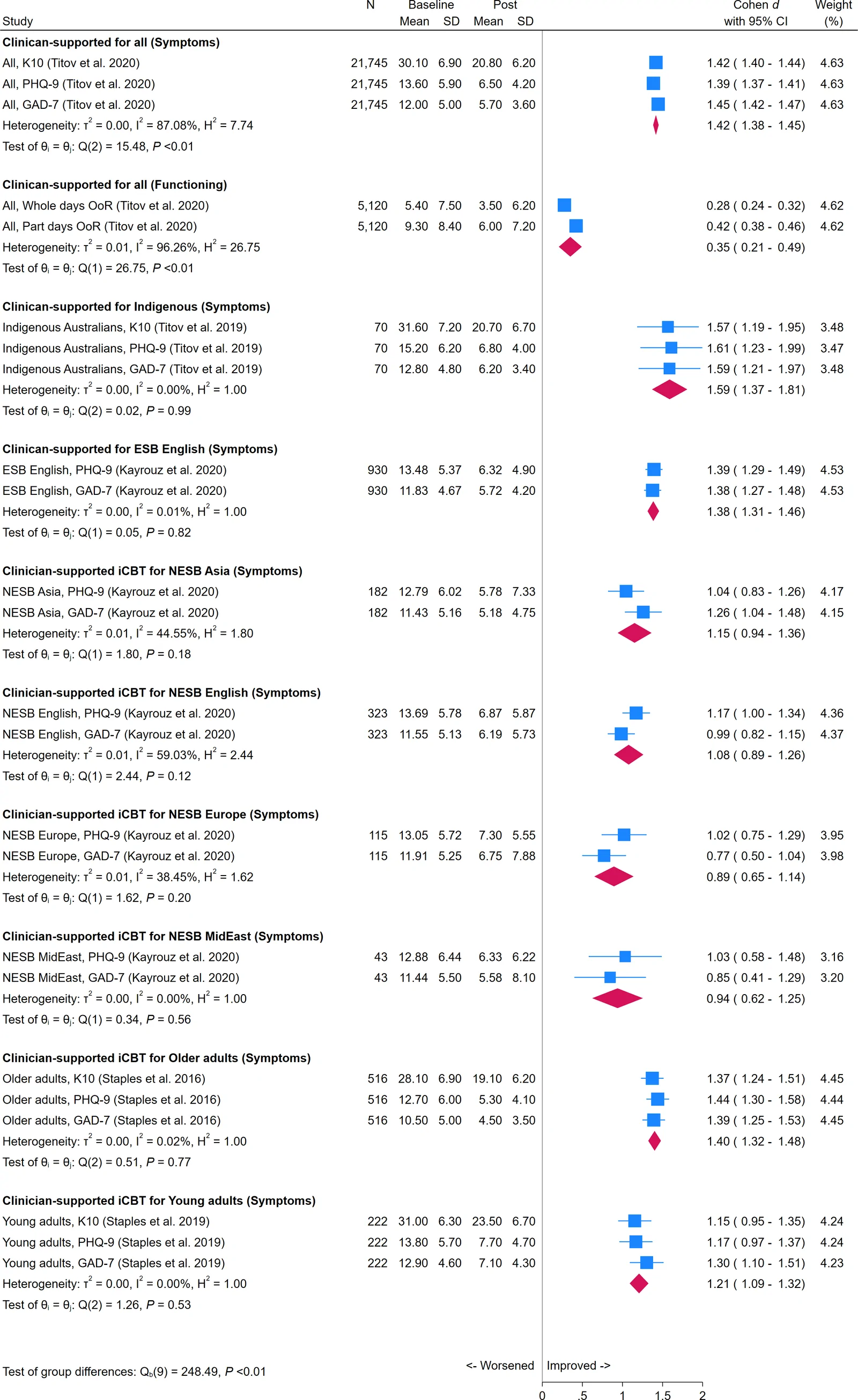
Forest plot of MindSpot (5 online cognitive behavioral therapy modules, clinician-supported) treatment effects on mental health symptoms and functioning, in all service users and by demographic characteristics [25,29-32]. ESB: migrant of an English-speaking background; GAD-7: Generalized Anxiety Disorder 7-item scale; iCBT: internet-based cognitive behavioral therapy; K10: Kessler Psychological Distress 10-Item Scale; NESB: migrant of a non–English-speaking background; OoR: out of role; PHQ-9: Patient Health Questionnaire-9.

**Figure 3.**
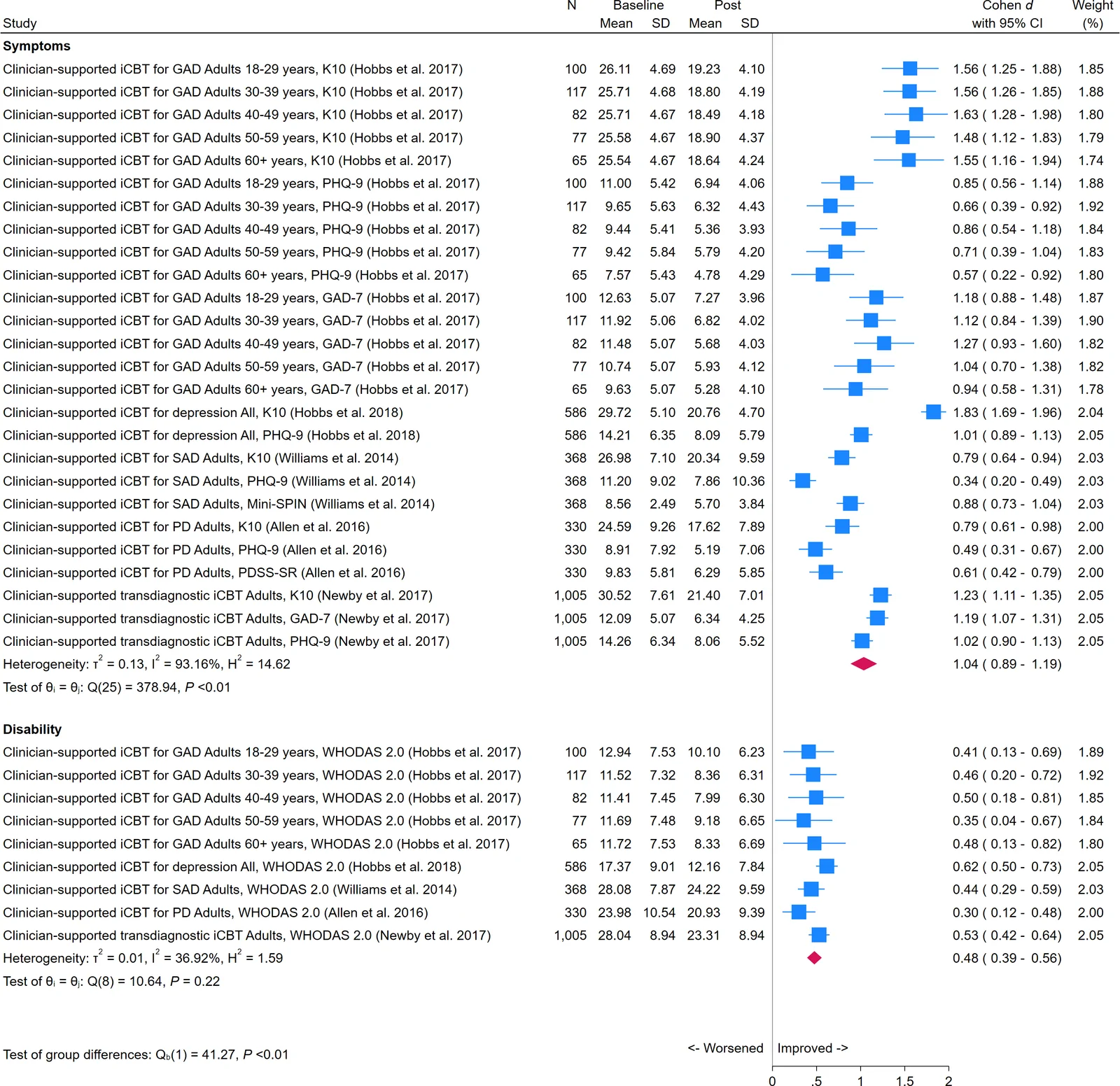
Forest plot of THIS WAY UP (6 online cognitive behavioral therapy modules, clinician-supported) treatment effects on mental health symptoms and disability [33-37]. GAD: generalized anxiety disorder; GAD-7: Generalized Anxiety Disorder 7-item scale; iCBT: internet cognitive behavioral therapy; K10: Kessler Psychological Distress 10-Item Scale; Mini-SPIN: Mini-Social Phobia Inventory; PD: panic disorder; PDSS-SR: Panic Disorder Severity Scale Self-Report version; PHQ-9: Patient Health Questionnaire-9; SAD: social anxiety disorder; WHODAS 2.0: World Health Organization Disability Assessment Schedule-II.

Figure 1 shows that Mental Health Online clinician-supported treatments produced large (Cohen *d*=0.95) and self-directed treatments produced medium (Cohen *d*=0.59) improvements in mental health symptom severity. However, treatment had small effects on quality of life irrespective of whether clinician-supported or self-directed (Cohen *d*=0.12 and Cohen *d*=0.28, respectively). There were significant differences in effect sizes between the 4 subgroups (*P*<.001). Studies in the same subgroup had similar effects (as demonstrated by low heterogeneity, with *I*^2^=0%‐35%; and no evidence against homogeneity, all *P*>.16).

Figure 2 shows that MindSpot clinician-supported treatments produced large to very large improvements in mental health symptom severity for the “all consumers” group. There are significant differences in effect sizes between the subgroups (*P*<.001), ranging from Cohen *d*=0.89 for migrants from non–English-speaking European countries to Cohen *d*=1.59 for Indigenous peoples. Additionally, clinician-supported treatment had small effects on functioning (Cohen *d*=0.35) as assessed by whole or part days out of role (using the K10+ [Kessler Psychological Distress 10 Plus-Item Scale]) [41]. The level of heterogeneity within subgroups varied substantially and ranged from approximately 0 (for Indigenous people, migrants from English-speaking backgrounds, migrants from the Middle East, older adults, and younger adults, all with low-moderate sample sizes) to extremely high (96% for the overall service user group for functioning). Because the sample sizes in the overall (all) service user groups were very large (>5000), confidence limits are very narrow, so the tests may be less relevant and useful for these groups. For example, the estimated effect sizes for mental health symptoms in the overall service user group were quite consistent, at Cohen *d*=1.42, Cohen *d*=1.39, and Cohen *d*=1.45. Heterogeneity was moderate at around 40%‐60% in subgroups with medium sample sizes, including migrants from Asia, non–English-speaking migrants from English-speaking countries, and migrants from Europe.

Figure 3 shows that THIS WAY UP clinician-supported treatments produced large (Cohen *d*=1.04) improvements in mental health symptom severity for adult consumers, across different age ranges, disorders (depression and anxiety disorders), and outcome measures. Clinician-supported treatment produced moderate effect size reductions in disability (Cohen *d*=0.48), which were similar across the adult lifespan and for different disorder types (depression and anxiety).

Significant differences were observed in pooled effect sizes between the mental health symptoms and disability subgroups (*P*<.001). High heterogeneity across age groups and between studies reporting on symptoms (*I*^2^=93% and *P*<.001) indicates differences in the magnitude of outcomes produced by THIS WAY UP clinician-supported treatment. This is partly due to the outcome measure, with larger effect sizes observed for the K10. Heterogeneity was much lower for the disability subgroup (*I*^2^=37% and *P*=.22), which means the age groups and studies in this subgroup produced outcomes of a similar magnitude.

### Effectiveness of DMHSs Compared With National and International Programs or Trials

Figure 4 presents effect sizes we calculated for each of the comparison treatments plotted against the pooled effect sizes we calculated for each of the DMHSs (0.95 for clinician-supported and 0.59 for self-directed treatment by Mental Health Online, 1.42 for clinician-supported treatment by MindSpot, and 1.04 for clinician-supported treatment by THIS WAY UP).

**Figure 4.**
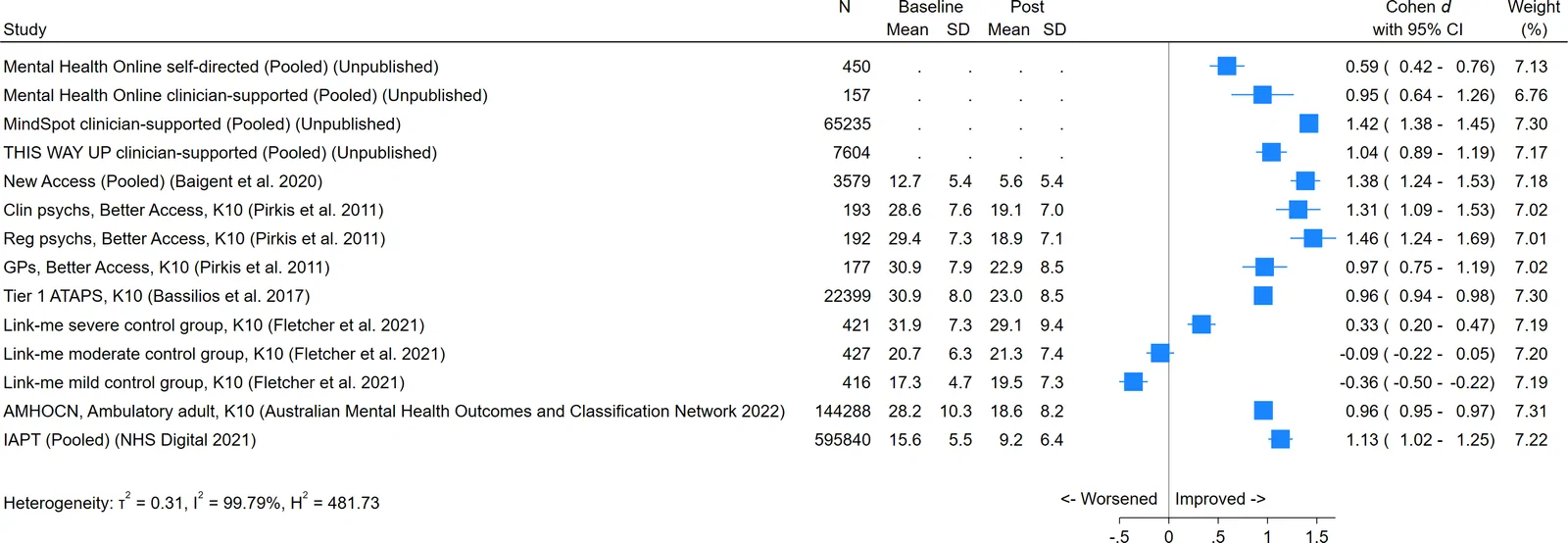
Forest plot of Cohen *d* (95% CI) in mental health outcomes for digital mental health services and comparison national and international programs or trials [22,41,46-49]. Pooled effect sizes for Mental Health Online treatment as per Figure 1 (“Clinician-supported (Symptoms)” and “Self-directed (Symptoms)”). Pooled effect size for MindSpot clinician-supported treatment as per Figure 2 (“All (Symptoms)”). Pooled effect size for THIS WAY UP clinician-supported treatment as per Figure 3 (“Symptoms”). Effect sizes are pooled for Patient Health Questionnaire-9 and Generalized Anxiety Disorder 7-item scale for New Access and IAPT. AMHOCN: Australian Mental Health Outcomes and Classification Network; ATAPS: Access to Allied Psychological Services (Tier 1 refers to base funding or general ATAPS vs Tier 2 funding for hard-to-reach groups); clin psychs: clinical psychologists; GP: general practitioner; IAPT: Improving Access to Psychological Therapies; K10: Kessler Psychological Distress 10-Item Scale; reg psychs: registered psychologists.

Most effect sizes were large, ranging from 0.95 (Mental Health Online and clinician-supported) to 1.46 (Better Access). Of these, the largest effect sizes (around 1.4) were observed for Better Access (delivered by registered psychologists), MindSpot (clinician-supported treatment), and New Access. One effect size was medium (Mental Health Online, self-directed, Cohen *d*=0.59) and 3 (all for Link-me) were small (ranging from −0.36 to 0.33). The Link-me effect sizes, which differ from all other services, are likely to have driven the observed heterogeneity. However, the aim of this study was to compare the magnitude of the effects between DMHSs and the comparators rather than to pool the effects across all included services.

## Discussion

### Summary of Findings

#### Overview

This study aimed to describe the uptake and effectiveness of 3 key Australian DMHSs that provide the option of clinician-supported online treatment. These DMHSs are providing treatment (among other service offerings) to significant numbers of consumers. Clinician-supported online treatment significantly improves the mental health of consumers, and self-directed treatment produces moderate reduction in symptom severity.

#### Uptake of DMHSs

Together, the 3 DMHSs have provided care to over 282,000 Australians between January 2013 and December 2021. Although this uptake is modest compared to around 1.3 million receiving at least one treatment session (with an eligible allied health professional) in 2021 through Australian Government–funded subsidized mental health care via the Better Access program [50], the DMHSs are contributing to improving overall access to mental health care in Australia. By offering their services free of charge and addressing other barriers to using face-to-face care, our larger mixed methods evaluation [10] showed that these DMHSs are reaching relatively significant proportions of typically hard-to-reach groups and/or groups who are less likely to seek help (eg, 7%‐19% from rural and remote locations, 4%‐8% Indigenous peoples, 9%‐29% males, 5%‐12% aged ≥55, and 22 % of MindSpot consumers are born overseas). Furthermore, findings from our overall evaluation of these DMHSs show that they are providing services to a high number of people who are not accessing any other support for their mental health [10]. Specifically, at least half of Mental Health Online and MindSpot consumers are not accessing other mental health services, which suggests DMHSs are reducing the burden of care on other, more resource-intensive, mental health services.

Our overall evaluation also showed a pronounced increase in registrations and enrollments for all 3 DMHSs examined during the COVID-19 pandemic, highlighting their high potential for scalability [10].

Over 80% of consumers who enroll in MindSpot and THIS WAY UP treatments commence treatment (equivalent data were not available from Mental Health Online). This suggests an efficient transition into treatment, with a minority of consumers disengaging from, or referred elsewhere for, treatment.

Adherence to treatment is as important a consideration as access to, or uptake of, DMHSs. We found that around two-thirds of those who started clinician-supported treatment with MindSpot and 46% of those who started clinician-supported treatment with THIS WAY UP completed treatment (ie, at least 4 of 5 lessons and two-thirds of lessons, respectively). The completion rate for MindSpot is comparable to, and for THIS WAY UP is lower than, UK published CBT completion rates (based on reason for discharge in IAPT) [51]. Treatment completion rates for THIS WAY UP self-directed and clinician-supported programs are similar, which is promising given that adherence to self-directed digital mental health programs is typically poorer [52].

However, this also indicates that around one-third of MindSpot and 54% of THIS WAY UP consumers drop out of clinician-supported treatment, which is higher than the 26% treatment dropout rate for CBT (delivered digitally or face-to-face to individuals or groups) reported in a meta-analysis, which found no significant differences between RCT and non-RCT studies [53]. More research is needed to determine reasons for dropping out of treatment because, in at least some cases, people may drop out due to improvement in mental health symptoms. For example, one study has shown that consumers who drop out of online courses benefit from each successive lesson completed to a similar degree as those who complete the entire course [54].

#### Effectiveness of DMHSs

Overall, we found that clinician-supported treatments by all 3 DMHSs produced improvements in mental health symptoms (Cohen *d*=0.95, Mental Health Online; Cohen *d*=1.42, MindSpot; and Cohen *d*=1.04, THIS WAY UP). Self-directed treatment by Mental Health Online produced moderate reduction (Cohen *d*=0.59) in clinical disorder severity ratings for generalized anxiety disorder (GAD), panic disorder, obsessive-compulsive disorder (OCD), posttraumatic stress disorder (PTSD), and social anxiety disorder (SAD).

Consistent with findings from our literature review [12], clinician-supported treatment produced reductions in psychological distress and symptoms of mental health disorders (OCD, PTSD, panic disorder, GAD, SAD, and depression). These positive findings are consistent across different demographic characteristics, including young and older adults, people born overseas, and Indigenous peoples. To some extent, these findings fill gaps in knowledge identified in our literature review about the effectiveness of DMHIs for traditionally underserviced groups and across age groups [12].

Clinician-supported treatment by the DMHSs also produced positive outcomes on quality of life, functioning (as assessed by days out of role), and disability, but the effects on these domains were smaller (eg, Cohen *d*=0.12, 0.35, and 0.48, respectively).

The magnitude of improvements in mental health symptoms produced by clinician-supported treatments was close or equivalent to most comparator treatments we examined (Australian primary, public, and low-intensity mental health care; UK stepped psychological care). Specifically, effect sizes ranged from Cohen *d*=0.95 for Mental Health Online to Cohen *d*=1.42 for MindSpot; and from Cohen *d*=−0.36 for the Link-me mild group receiving no or usual treatment to Cohen *d*=1.46 for treatment by registered psychologists via Better Access. Both clinician-supported and self-directed treatments produce superior outcomes to treatment as usual (discussing mental health concerns with the general practitioner [GP]). They also produce superior outcomes to pharmacological treatment in primary care, which has been reported to produce small to moderate effects [25].

However, there was heterogeneity in the magnitude of improvements produced by the 3 DMHSs and comparator treatments. Heterogeneity was expected given the diversity (in populations, service design and delivery, implementation contexts, outcome measures, and treatment lengths) of both the 3 DMHSs evaluated and the comparator interventions, which ranged from low-intensity interventions for mild mental health needs to high-intensity services targeting more severe presentations. Although service-level pooled estimates provide a useful summary of the overall direction of effects, the observed variability suggests findings should be interpreted with caution and highlights the importance of considering for whom, and in what circumstances, DMHSs are most effective.

Nonetheless, the positive effects on mental health were maintained over time, with MindSpot showing that consumer benefits were maintained 3 months post treatment [25]. Finally, consumers were highly satisfied with the care they received from these DMHSs, with MindSpot and THIS WAY UP routinely collected data showing that between 81% and 98% of consumers reported treatment was worthwhile, and they would recommend it [10].

### Limitations

Our findings should be interpreted in the context of several caveats. Most of our effectiveness findings were based on analysis of secondary data we extracted from purposively selected key peer-reviewed publications from the wealth of studies published by the DMHSs. However, it was beyond our scope to extract data from all their publications. Even if we used deidentified individual-level consumer data, some data elements are either lacking or not readily available in the data capture systems of the DMHSs (eg, nature and dose of clinician support for all 3 DMHSs and posttreatment outcome data for Mental Health Online are not captured). For example, around one-third of MindSpot consumers who enroll in clinician-supported treatment choose not to take up the clinician support component, and around one-third of consumers who enroll in self-directed treatment end up receiving clinician support, but the data system only captures clinician support at enrollment (N Titov, personal communication, March 15, 2022). Similarly, although in our pooled estimates of THIS WAY UP treatment effects, we labeled all treatments as “clinician-supported,” the extent of clinician support is unknown given that this service element is provided externally by the consumer’s own mental health professional. One study of THIS WAY UP reported the median number of clinician contacts was 1, and more than 50% of patients reported that they had no contact from their clinician while completing their online course [45]. Therefore, differences in findings between clinician-supported and self-directed treatments should be interpreted with caution.

Similarly, because of the real-world nature of the evaluation and the limited time frame available to us, it was not feasible to include a comparison group. As a result, we relied on published findings of diverse comparison mental health treatments and treatment-as-usual control groups who may not be representative of consumers using DMHSs.

Certainty and generalizability of effectiveness estimates are limited by complete-case analysis (excluding participants without pre-post outcome data) and heterogeneity of the pooled effects of each DMHS and comparator treatment. However, heterogeneity was expected given the variability across both the DMHSs and the comparator interventions—spanning a broad range of intensities and levels of mental health need.

Finally, despite the achievements of the 3 DMHSs, their impact has been reported in isolation from community needs analyses, prohibiting comment on the true magnitude of their achievements or their reach within the intended target consumer groups.

### Strengths

A key strength of this study is that it was conducted as one component of a larger evaluation that used multiple additional data sources including an environmental scan [11], an umbrella review [12], and consultations with a large number and broad range of stakeholders (consumers [13], DMHS providers, people with lived experience of mental health problems with or without experience using DMHSs, additional health professionals with or without experience using DMHSs such as GPs and mental health professionals, and other key mental health sector stakeholders) [10]. This enabled us to assess processes and impacts and to triangulate findings from a range of perspectives. Our collaborative approach with the 3 DMHSs and the Australian Department of Health (now the Department of Health, Disability and Ageing) optimizes the potential utilization of our findings.

### Implications and Future Research

It is recommended that Australia and other countries with similar health systems (ie, universal public coverage with a parallel private system) foster clinician-supported DMHSs as a treatment option by increasing funding to expand their availability, commissioning ongoing evaluation of their performance, and developing information campaigns for people with lived experience of mental illness and health professionals to increase awareness of clinician-supported DMHSs [8]. It is essential to understand how DMHSs work and sit in the broader mental health service system, particularly in contexts such as the COVID-19 pandemic in which traditional face-to-face services may not be tenable. Multipronged, innovative approaches may facilitate embedding DMHSs in the mental health system. Examples include collaborating and partnering with other parts of the mental health system and tertiary education, or developing on-site spaces for consumers to access DMHSs (eg, health clinics and other face-to-face health and community services) to improve access to DMHSs in low socioeconomic and rural and remote areas where internet connections may be unreliable or unaffordable.

Efforts to enhance and standardize data collection and outcome measurement across services and embedding evaluation as part of a continuous improvement approach within DMHSs will contribute to building the evidence base and improving our understanding of the role of clinician-supported DMHSs in the mental health service system. Evaluation of DMHSs should be ongoing, and findings shared to allow scaling up of service models, or components of service models which are effective. Standardized data collection and outcome measurement will improve the robustness of statistical analysis and maximize comparisons between DMHSs and other areas of the mental health system.

For example, data on dose and nature of clinician support may provide important insights about adherence and effectiveness, but these data are not captured in the existing data repository systems of the services we examined. Our review of reviews highlighted diversity in the type, format, and extent of support or guidance offered; and in the duration of treatment [12].

Assessing consumer outcomes at the commencement and the completion of treatment risks introducing a systematic bias in which people who drop out of treatment (potentially with poorer outcomes) are excluded from effectiveness analyses. However, a key strength of the way in which outcome data are collected by MindSpot and THIS WAY UP is that consumers are assessed using standardized outcome measures at each session. This means that analysis of their outcome data provides the opportunity to examine the effects of treatment for consumers who drop out of treatment.

Although robust evidence exists for the efficacy and effectiveness of using DMHIs to treat depression and anxiety in adults [12], further research is needed to explore the efficacy of these interventions for young people and traditionally underserviced groups (eg, Indigenous peoples, people from culturally and linguistically diverse backgrounds, and people who are LGBTQIA+); and other mental disorders (eg, psychotic disorders, personality disorders, and substance dependence) and comorbid conditions. For example, exploring whether consumers experiencing psychotic disorders, suicidal ideation, and/or more complex presentations benefit from clinician-supported DMHS treatment as a component of a suite of care (eg, medical follow-up and specialist mental health care), which for many does not include any evidence-based psychological treatment [55]. Additionally, the proportion of recovered or improved consumers postintervention should be considered in future studies since our study focused on average effects across samples.

Our environmental scan of DMHSs indicated that they are not suitable or the preferred choice for everyone, with some consumers preferring face-to-face services [11]. It also highlighted many opportunities for improving reach and use of DMHSs, making optimal use of technology, and embedding and sustaining DMHSs in the mental health service system. Therefore, future research is also needed to increase understanding of who engages with, adheres to, and benefits from DMHSs, and why. This work could contribute to comprehensive assessment and screening of consumers to identify consumers suited to and most likely to benefit from DMHSs. It may also help identify which people do not engage with DMHSs and how this might be addressed. Finally, notwithstanding the importance of clinician support in providing effective DMHSs, the role of technological advances, such as AI, in personalizing or tailoring DMHS delivery warrants consideration to address differences in consumer needs, promote adherence and positive outcomes, and potentially increase efficiency.

### Conclusions

This study has shown that Mental Health Online, MindSpot, and THIS WAY UP provide consumers with a range of service offerings including assessment and treatment. They have provided these services free of charge to a substantial number of adult consumers and have become an integral part of Australia’s mental health care system. The outcome data, where available, show that treatments delivered by DMHSs are producing significant clinical improvement for consumers. The magnitude of improvement produced via clinician-supported treatment is comparable with more resource-intensive face-to-face treatment options. These services have the potential to be scalable and good value for money.

The DMHSs are largely intended to target consumers with depression and anxiety disorders who choose to use digital mental health treatment or who, for a range of reasons, may have limited or no access to alternative treatment options. DMHSs are not intended to serve Australia’s entire help-seeking population, which may be better served through other components of the mental health system (eg, the larger-scale Better Access program, state-funded public mental health services, and the not-for-profit sector). DMHSs are relatively new innovative elements of the Australian mental health care landscape and will become further embedded with time. DMHSs are contributing to ensuring that consumers get the right care at the right time and, importantly, in accordance with consumer needs and preferences.

## Supplementary material

10.2196/90121 Multimedia Appendix 1 Mental health and other outcome measurement scales used by digital mental health services.

## Abbreviations

CBT: cognitive behavioral therapy
DMHI: digital mental health intervention
DMHS: digital mental health service
GAD: generalized anxiety disorder
GAD-7: Generalized Anxiety Disorder 7-item scale
GP: general practitioner
IAPT: Improving Access to Psychological Therapies
K10: Kessler Psychological Distress 10-Item Scale
K10+: Kessler Psychological Distress 10 Plus-Item Scale
K6: Kessler 6-item version
OCD: obsessive-compulsive disorder
PHQ-9: Patient Health Questionnaire-9
PTSD: posttraumatic stress disorder
RCT: randomized controlled trial
RE-AIM: Reach, Efficacy or Effectiveness, Adoption, Implementation, and Maintenance
SAD: social anxiety disorder
