# Supplementary material for: Australian Digital Mental Health Services for Anxiety and Depression: Meta-Analysis and Comparison Study
Source: JMIR Form Res. 2026 Oct 6;10:e90121. doi: 10.2196/90121 (PMC13641322; doi:10.2196/90121)
Supplement: Supplementary file 1 [file formative-v10-e90121-s001.docx]

## Multimedia Appendix 1: Mental health and other outcome measurement scales used by DMHSs

| Measure | Brief description | Scoring |
| --- | --- | --- |
| Generalised Anxiety Disorder scale (GAD-7) [17] | Consumer-rated measure of anxiety symptoms. Comprises seven questions about how often the consumer has been bothered by selected anxiety symptoms in the past two weeks. | Each item is scored 0 (Not at all), 1 (Several days), 2 (More than half the days) or 3 (Nearly every day). The total score ranges from 0–21. A score of 10 or more indicates the likely presence of Generalised Anxiety Disorder. |
| Kessler Psychological Distress 10-Item Scale (K10) [15] | Consumer-rated measure developed to assess non-specific psychological distress. Comprises 10 items about symptoms of depression and anxiety in the past four weeks. | Each item is rated from 1 (None of the time) to 5 (All of the time), resulting in a total score that ranges from 10 to 50. Scores of 10–15 indicate little or no psychological distress, scores of 16–21 indicate moderate psychological distress, scores of 22–29 indicate high psychological distress, and scores of 30–50 indicate very high psychological distress. |
| Kessler Psychological Distress 10 Plus-Item Scale (K10+) [38] | As described above and includes four additional questions to assess degree of disability experienced by consumer. | The additional four questions do not contribute to the total score. They provide number of full or part days out of (work/education) role and number of visits to health services. |
| Kessler Psychological Distress 6-Item Scale (K6) [18, 19] | Consumer-rated measure of non-specific psychological distress intended to be used as a quick tool to assess risk for serious mental illness in the general population. Comprises six items about symptoms of depression and anxiety in the past four weeks. | Each item is rated from 1 (None of the time) to 5 (All of the time), resulting in a total score ranging from 6 to 30. Scores of 6–18 indicate no probable serious mental illness and scores of 19–30 indicate probable serious mental illness. |
| Mini-Social Phobia Inventory (Mini-SPIN) [39] | Consumer-rated three-item scale about avoidance and fear of embarrassment over the past week to screen for generalised social anxiety disorder. Developed based on the longer 17-item social phobia inventory (SPIN). | Each item is rated using a 5-point Likert scale from 0 (Not at all) to 4 (Extremely). Scores range from 0 to 12, with scores of 6 or higher indicating possible problems with social anxiety. |
| Patient Health Questionnaire (PHQ-9) [16] | Consumer-rated measure of depressive symptoms. Comprises nine items about how often the consumer has been bothered by depressive symptoms in the past two weeks. | Each item is scored 0 (Not at all), 1 (Several days), 2 (More than half the days) or 3 (Nearly every day). Total scores range from 0 to27. Scores of 0–4 indicate no depression, scores of 5–9 indicate mild depression, scores of 10–14 indicate moderate depression, scores of 15–19 indicate moderately severe depression, and scores of 20–27 indicate severe depression. |
| Posttraumatic Stress Disorder Checklist (PCL-C) [40] | Consumer-rated 17 item measure that assesses the DSM-5 symptoms of PTSD over the past month. | Items are rated on a five-point scale, from 1 (not at all) to 5 (extremely) and generate a total symptom severity score between 17 and 85. Scores of 17–29 indicate little to no severity; scores of 30–44 indicate moderate to moderately high severity; and scores of 45–85 indicate high severity. |
| Panic Disorder Severity Scale Self-Report version (PDSS-SR) [41] | Consumer-rated seven-item measure of panic disorder symptoms over the previous week. | Items are rated on a five-point scale from 0 (not at all/none) to 4 (extreme) and generate a total score between 0 and 28. Cut-off scores ≥8 suggest clinical levels of panic disorder. |
| World Health Organization Disability Assessment Schedule (WHODAS 2.0) [42] | Consumer-rated 36-item measure that assesses health and disability across six domains including: measured functional impairment.  Consumer-rated 12-item measure of functional impairment and activity limitation over the past 30 days. Assesses Cognition, Mobility, Self-care, Getting along, Life activities and Participation. | Items are assessed on a five-point scale, from 1 (none) to 5 (extreme) and are summed to yield a score ranging from 12 to 60. Higher scores indicate higher disability or loss of function. |
| World Health Organization Quality-of-Life Scale (WHO-QOL-BREF) Psychological Domain subscale [43] | Consumer-rated 26-item instrument consisting of four domains: physical health (7 items), psychological health (6 items), social relationships (3 items), and environmental health (8 items); it also contains QOL and general health items. The psychological domain measures include self-image, negative thoughts, positive attitudes, self-esteem, mentality, learning ability, memory concentration, religion, and mental health status. | Each item is scored on a five-point scale, from 1 (not at all) to 5 (completely), to determine a raw score. Mean score of items in each domain is used to derive each domain’s score. Higher scores indicate better quality of life. |
| Yale Brown Obsessive-Compulsive Scale (Y-BOCS) [44] | Clinician-administered 10-item scale used to assess for obsessive compulsive disorder | Items are scored on a five-point scale from 0 (no symptoms) to 4 (extreme symptoms). The sum of the first five items is a severity index for obsessions, and the sum of the last five is an index for compulsions. Scores of 0–7 indicate subclinical symptoms; 8–15 indicate mild symptoms; 16–23 indicate moderate symptoms; 24–31 indicate severe symptoms; and 32–40 indicate extreme symptoms. |
